# The Effect of Amifostine on Submandibular Gland Histology after Radiation

**DOI:** 10.1155/2012/508279

**Published:** 2012-07-15

**Authors:** Jacqueline C. Junn, James J. Sciubba, Justin A. Bishop, Eva Zinreich, Mei Tang, Marshall A. Levine, Robert A. Palermo, Carole Fakhry, Ray G. Blanco, John R. Saunders, Joseph A. Califano, Patrick K. Ha

**Affiliations:** ^1^New York Medical College, Valhalla, NY 10595, USA; ^2^The Milton J. Dance, Jr. Head and Neck Center, Greater Baltimore Medical Center, Baltimore, MD 21204, USA; ^3^Department of Pathology, Johns Hopkins University School of Medicine, MD 21205, USA; ^4^Department of Radiation Oncology, Greater Baltimore Medical Center, Baltimore, MD 21204, USA; ^5^Department of Medical Oncology, Greater Baltimore Medical Center, Baltimore, MD 21204, USA; ^6^Department of Pathology, Greater Baltimore Medical Center, Baltimore, MD 21204, USA; ^7^Division of Head and Neck Cancer Surgery, Department of Otolaryngology-Head and Neck Surgery, Johns Hopkins University School of Medicine, Baltimore, MD 21231, USA

## Abstract

*Background*. The purpose of this study was to assess the effects of amifostine on submandibular gland histology in patients receiving chemoradiation therapy. *Methods.* We conducted a retrospective submandibular gland histologic slide review of HNSCC patients receiving chemoradiation for head and neck squamous cell carcinoma with three different levels of amifostine exposure. We used six scoring parameters: fatty replacement, lobular architecture degeneration, interstitial fibrosis, ductal degeneration, acinar degeneration, and inflammatory component presence. *Results*. Differences in gender, tumor stage, amifostine dose, age, number of days after neck dissection, and smoking history (pack years) exposure were not significant between the three groups, although there was a difference between groups in the primary subsite (*P* = 0.006). The nonparametric Cuzick's test for histologic parameters with varied amifostine treatment showed no significance among the three groups. *Conclusions*. Although patients did not receive a full dose of amifostine due to side effects, varying doses of amifostine had no apparent evident cytoprotective effects in three groups of cancer patients treated with primary chemoradiation.

## 1. Introduction

Head and neck squamous cell carcinoma (HNSCC) is the sixth most common cancer in the United States [1] and includes cancers of the paranasal sinuses, oral cavity, nasopharynx, oropharynx, hypopharynx, and larynx. Combined chemoradiation therapy is one commonly employed treatment option for head and neck cancer patients with advanced stage disease. Salivary glands often lie within the radiation field with resultant radiation damage leading to undesirable functional sequelae, such as xerostomia [2]. Because xerostomia has distressing consequences on patients' lives [3, 4], developing protective mechanisms for the salivary glands is an important avenue of investigation. Amifostine is one such radioprotective drug currently approved for use to reduce the occurrence of xerostomia in head and neck cancer patients by way of providing a reduction of radiation-related damage to salivary gland parenchyma.

Amifostine is administered intravenously or subcutaneously as an inactive prodrug. Alkaline phosphatase converts amifostine into an active thiol by dephosphorylation [5]. The cytoprotective selectivity of amifostine is based on the differences between the physiological environment of normal and tumor cells; tumor cells are hypovascular, have low interstitial pH, and lower expression of alkaline phosphatase than normal cells [6]. Hence, amifostine acts preferentially on normal cells, rather than tumor cells. Once activated, it accumulates within the cells and confers its protection as a scavenger by eliminating free radicals, upholding membrane integrity, and preventing DNA damage.

Previously, human clinical trials and animal studies have shown amifostine as a promising radioprotective agent that may reduce xerostomia in patients with acute and chronic xerostomia [7], and preserve salivary gland function, especially of the parotid glands [8, 9]. However, it also had clinically undesirable manifestations, such as thrombocytopenia and leucopenia [10]. In animal studies, amifostine demonstrated a protective effect on the organ of Corti in irradiated guinea pigs [11] and reduced parenchymal damage of salivary glands in rabbits [12].

Although many randomized cohort studies have demonstrated amifostine's ability to protect noncancerous cells, the actual benefit remains controversial. Currently, the therapeutic effects of amifostine remain debatable due to the inconsistencies among various clinical studies [13, 14]. For instance, whereas some patient studies have claimed significant benefit [7, 15, 16], others only demonstrated restricted relief of xerostomia [17–19]. To better understand the actual morphological impact of amifostine, histological examination of human salivary gland samples is needed.

Thus far, no studies have examined the histological differences within salivary glands in head and neck cancer patients treated with amifostine. The aim of this study was to evaluate the morphological manifestations of amifostine on patients, and we therefore conducted a histological study of the submandibular glands of 24 head and neck patients undergoing primary chemoradiation therapy.

## 2. Materials and Methods

### 2.1. Clinical Samples

Twenty-four submandibular gland samples were retrospectively collected based on specimen availability from a cohort of 108 patients undergoing a uniform, primary chemoradiation regimen at the Greater Baltimore Medical Center. All patients were previously untreated and received twice daily radiation of 125 cGy five days a week as a split course over a 45-day-period. This included concomitant cisplatin (12 mg/m^2^/1 h) and 5-fluorouracil (600 mg/m^2^/20 h) on days 1 through 5 and 29 through 33. A total dose of 7000 to 7500 cGy was targeted to the primary tumor site; 6000 cGy was targeted to involved lymph nodes; 5000 cGy was targeted to uninvolved lymph nodes. A treatment break of one week was included following 4000 cGy. The study was approved by the GBMC IRB number 08-025-07. All patients examined in this study had stage IV disease, with their submandibular glands uninvolved by tumor. All patients received conventional radiation therapy at a dosage calculated to be above 5940 cGy to the submandibular gland regardless of the primary tumor location. They received a routine neck dissection including the submandibular gland at an average of twelve weeks after the end of their treatment.

Patients were divided into three groups: amifostine, partial-amifostine, and no amifostine. They were given the option to include amifostine in their treatment regimen. The amifostine group and partial-amifostine group received a daily dose of 500 mg of amifostine intravenously per dose given concurrently with radiation. Administration of amifostine was recorded as outpatient therapy. The amifostine group received 10 or more doses, the partial-amifostine group received less than 10, and the nonamifostine group received none. Patients who received only partial dosing generally stopped due to drug-related side effects, primarily nausea.

### 2.2. Histological Examination

The slides were prepared by the Department of Pathology at Greater Baltimore Medical Center using standard hematoxylin and eosin staining. The histological examination was conducted twice by two board-certified head and neck pathologists (J. J. Sciubba and J. A. Bishop) who independently rated each slide in a blinded fashion. They came to a consensus grading scale after their initial independent examination. The histological parameters were semiquantitatively defined by essential histological features, including the degree of fatty replacement, preservation of lobular architecture, preservation of ducts and acini, the presence of interstitial fibrosis, and the inflammatory component. The scales used were no degeneration (0), trace (1), mild (2), moderate (3), and severe (4) degeneration.

### 2.3. Data and Statistical Analysis

This study was a pilot study; therefore, the study population represents a convenience sample. The study was designed to validate the histological scoring schema which is novel and had not previously been implemented. Given these restraints, assumptions of expected differences in distribution of histologic scores were not made a priori. The primary statistical objective of this study was to determine if the pathological grade of submandibular gland histologic features was correlated with the extent of amifostine treatment a patient received—≥10 doses of amifostine, <10 doses of amifostine, or no amifostine. The non-parametric Cuzick's test for trend [20] was used to compare the grades of fatty replacement, loss of lobular architecture, presence of interstitial fibrosis, degree of ductal degeneration, presence of diffuse inflammatory component, presence of focal inflammatory component, and degree of acinar degeneration across these three groups. Mean age, days since the end of treatment before neck dissection, and pack years of smoking were compared by dose groups with linear regression models. Comparison of gender, stage, and site between the treated and untreated patients were made with the Fisher's Exact test. All statistical computations were performed using the SAS system [21], StatXact [22], or R [23]. All *P* values reported are two-sided.

## 3. Results

### 3.1. Demographic Variables

Relevant patient demographic information is represented in Table 1. The mean age, number of days from the end of treatment to neck dissection, and pack years of smoking were not significantly different from the three treatment groups.

When we combined the full-amifostine and partial-amifostine groups (treated groups) and compared that to the no-amifostine group (untreated group), males made up 79% of the treated groups and 80% of the untreated group. All of the full-amifostine and partial-amifostine patients had tumors in the oropharynx whereas 50% of the untreated patients had the oropharynx as the primary tumor site with the other 50% located in the hypopharynx and larynx, *P* = 0.006. As the patients received very consistent radiation doses to the neck across subsites, we believe that the primary tumor site should have little effect on the findings.

### 3.2. Histological Parameters

The nonparametric Cuzick's test for trend was used to determine whether or not histologic parameters varied with the amount of treatment received. The findings are summarized in Tables 2 and 3. The trends were not significant for all six parameters. The *P*-values are as follows: degree of fatty replacement (*P* = 0.82), loss of lobular architecture (*P* = 0.57), presence of interstitial fibrosis (*P* = 0.26), degree of ductal degeneration (*P* = 0.19), presence of diffuse inflammatory component (*P* = 0.55), presence of focal inflammatory component (*P* = 0.94), and degree of acinar degeneration (*P* = 0.35).

**Figure 1:**
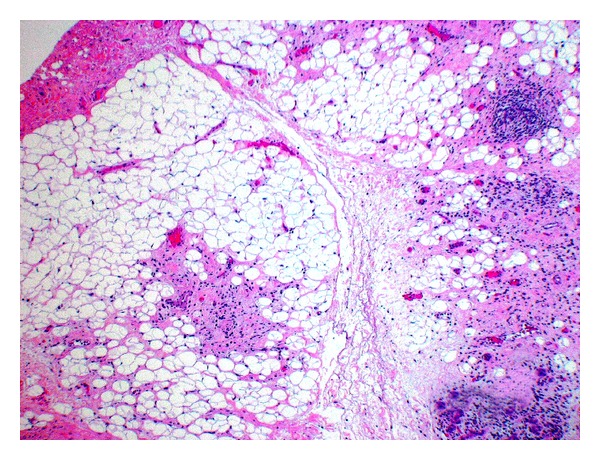
No-amifostine group (HE, Original magnification ×4.8). Near total fatty replacement of salivary lobules with focally intense lymphocytic inflammation can be observed. Note the effacement of general lobular architecture.

**Figure 2:**
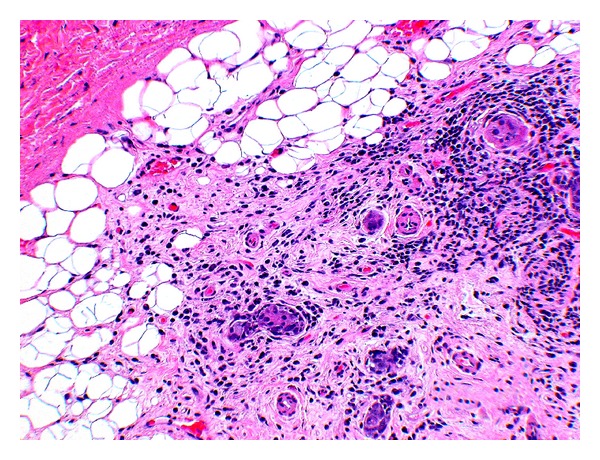
No-amifostine (HE, Original magnification ×28). The submandibular ductal remnants are within areas representing interstitial fibrosis as well as diffuse and focal lymphocytic infiltration.

Histological examination sections from the three groups showed mild to moderate fatty replacement of normal glandular architecture. The worst representation or highest level of fatty replacement was seen in the no-amifostine group with near total fatty replacement of salivary lobules and focally intense lymphocytic inflammation (Figure 1). In contrast to the severe fatty replacement observed in all three groups, lobular architecture was more consistently preserved. Even with overall preservation of lobular structure, samples from all three groups showed moderate loss of lobular architecture where the submandibular ductal remnants were surrounded by interstitial fibrosis and lymphocytic infiltrates (Figure 2).

**Figure 3:**
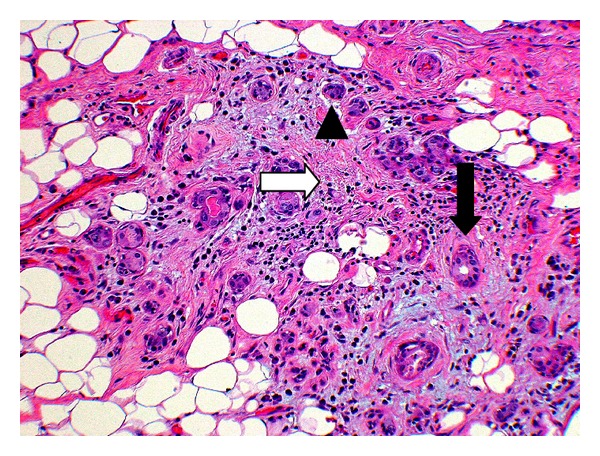
Partial-amifostine (HE, Original magnification ×28). Submandibular gland with partial-amifostine treatment reveals interstitial fibrosis (white arrow), scattered acinar remnants (arrowhead), and intact ductal remnants (black arrow).

All three groups showed marked interstitial fibrosis, present in 83% of our samples. We saw comparable amounts of diffuse, periductal, and periacinar interstitial fibrosis in all three groups. The sample from the partial-amifostine treatment group showed severe interstitial fibrosis that surrounded scattered acinar remnants and ducts (Figure 3). Interstitial fibrosis was also observed surrounding branching intralobular ducts (not pictured) and near fatty replacement (Figure 2).

**Figure 4:**
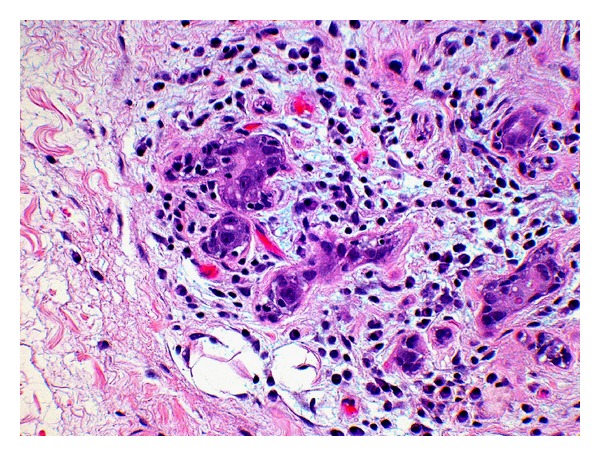
No-amifostine (HE, Original magnification ×28). Submandibular gland of no-amifostine patient shows lobular remnants and residual ducts with vacuolar degeneration. Ducts are surrounded by lymphoplasmacytic infiltrate and mild fibrosis.

**Figure 5:**
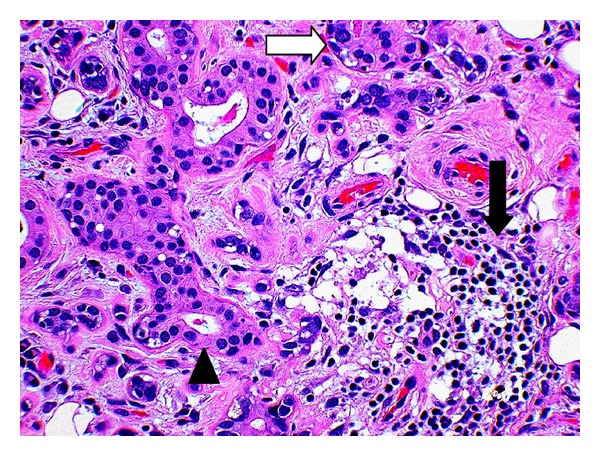
Amifostine (HE, Original magnification ×28). Acini show atrophy (white arrow) with duct preservation (arrowhead), and fibrosis and focal inflammation (black arrow).

Salivary ducts were more preserved than the acini in all three groups. Overall, the ducts maintained their normal architecture, but some of the lining cells appeared vacuolated, while others ducts were surrounded by lymphocytic infiltrates and fibrosis (Figure 4). About 63% of the samples showed moderate to severe acinar degeneration. Atrophied acini with focal, chronic inflammation could be detected (Figure 5). Furthermore, scattered acinar remnants could be seen throughout the collected samples in all three groups.

**Figure 6:**
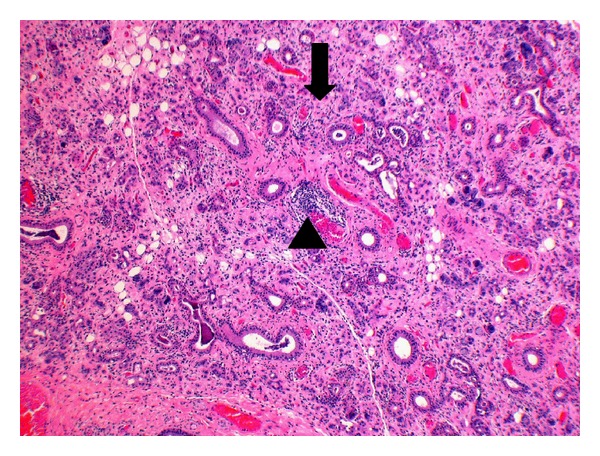
No-amifostine (HE, Original magnification ×4.8). Submandibular gland of no-amifostine patient reveal diffuse (black arrow) and focal chronic inflammation (arrowhead), acinar atrophy, diffuse fibrosis and dilation of ductal remnants.

We observed both diffuse and focal inflammatory components in the three groups. Overall, diffuse inflammatory components (75% of total samples) were more prominent than focal inflammatory components (38% of total samples). A sample taken from the no-amifostine group showed the most diffuse and focal inflammation. The submandibular gland revealed diffuse and focal chronic inflammation, acinar atrophy, and dilation of ductal remnants (Figure 6). These inflammatory components were mostly lymphoplasmacytic, illustrating its chronicity.

## 4. Discussion

Patients often develop debilitating side effects following chemoradiation therapy to the head and neck area, which impair overall function and quality of life. Prophylactic approaches have been implemented to minimize these undesirable sequelae. One approach includes the use of pharmacological agents, such as amifostine, which has been shown to reduce the clinical manifestation of radiation-related xerostomia. No studies have investigated the histological correlate of any protective ability of amifostine on human salivary tissue samples thus far. Our study was designed to evaluate the histological effects of amifostine on salivary tissue in patients undergoing multimodality therapy for head and neck squamous cell cancer.

We evaluated six histological parameters: degree of fatty replacement, interstitial fibrosis, degeneration of lobular architecture, ducts, acini, and the presence of an inflammatory component. Our results show an unremarkable protective function of amifostine in irradiated submandibular gland tissue, regardless of the levels of amifostine exposure and dosing. From the histological criteria used, fatty replacement, acinar loss, interstitial fibrosis, and diffuse inflammatory components were well pronounced across all three groups. Although age is a factor in increasing the presence of fat in salivary glands [24], the treatment groups in our study were comparable with respect to this factor and other demographics so as not to confound our findings.

As Sagowski et al. have alluded to in their analysis [25], previous animal studies did not reflect clinical scenarios because these animals received single doses of radiation and amifostine. In their study, the mice were irradiated with 60 Gy for 6 weeks with or without 250 mg/m^2^ of amifostine. The authors concluded that amifostine did not protect the salivary glands from acute radiation damage. Both arms showed progressive vacuolar alterations in acinar and ductal epithelial cells with interstitial edema also present. However, they reported a potential long term benefit in that the treated mice developed less fibrosis and necrosis.

Münter et al. recently reported that amifostine prevented the loss of salivary gland function when the patients received radiation doses less than 40.6 Gy [18]. Because damage to the salivary glands is dose dependent, the limited effect of amifostine in our patient population could be due to irradiation at a higher but therapeutic dose. In Rudat et al.'s study [9], they rescinded their earlier findings published in Münter et al. [18], which stated that patients irradiated at greater than 50 Gy showed no benefit with amifostine treatment three months after their radiation therapy. From Rudat et al.'s follow-up study, they observed statistically significant parotid gland function one year after radiation therapy ended. Their new finding does not contradict our study because our samples were obtained on average 86 days after the end of our patient's treatment regimen. Our findings are in line with their earlier study, which was of shorter duration as well. We chose our date of examination as it coincided with the routine surgical removal of the gland during planned neck dissection and thus concur that it is possible to have seen differences in our patients if we followed up in a long-term study. In addition, a recent study by Haddad et al. reported that patients receiving intensive chemoradiation therapy regimen did not show a clinically evident decrease in the incidence of xerostomia and mucositis with the use of amifostine [19]. Their findings question the possible added benefit of amifostine in patients undergoing intensive chemoradiation therapy.

While this was a retrospective review of a nonrandomized, limited patient population, it is the first to investigate the effects of amifostine in human submandibular glands in patients undergoing chemoradiation therapy. Our goal was to examine the histopathology of the glands rather than to correlate the morphology with the function of the glands with or without amifostine exposure.

The lack of difference among the groups could be attributed to the incomplete dosing of amifostine due to toxicity. Studies have suggested that in order to see clinical protective effects of amifostine, it should be administered 30 minutes before each radiotherapy or chemotherapy [7, 15, 26]. Our patients underwent twice daily radiation, but received only once daily dosing of amifostine due to nausea, vomiting, and hypotension. Twice-a-day dosing of amifostine would have imposed too much physical discomfort to patients and possibly have led to treatment interruption. In addition, the lack of noted difference between groups could also be due to the lack of power due to a small sample size. We acknowledge the limitations of this study in our methodology by using a non-parametric test of difference. Furthermore, even though our study was not randomized and had a limited number of patients, it is unlikely that the distribution of our demographic variables or treatment variability would bias the histological findings.

## 5. Conclusion

In conclusion, amifostine had no evident cytoprotective effect in patients with varying levels of amifostine exposure from our analysis using six histological parameters, though. It is unclear whether a poor histological appearance translates into a worse functional result. Thus far, the benefit of amifostine as a cytoprotective agent remains controversial. Our results are preliminary in that patients did not receive the full recommended amifostine regimen, and it is possible that more evident protective effects at the histological level would be seen with the higher total dose. Further research should investigate the relationship between the histological findings and the clinical outcomes in a long-term setting.

## Figures and Tables

**Table 1:** Patient demographics.

Parameter	Amifostine	Partial-amifostine	No-amifostine	*P*-value
Number	5	9	10	
Mean age	60	58	58	0.87^1^
95% confidence interval	45.5–74.1	52.3–63.1	53.6–62.0	
Range	45–70	50–71	48–68	
Mean days since end of treatment	88	89	81	0.85^1^
95% confidence interval	39.1–137.1	68.5–110.4	58.2–105.0	
Range	46–134	55–151	46–139	
Mean amifostine dose	17	5	0	0.0001^1^
95% Confidence Interval	13–21.8	3.7–7.2	0	
Range	12–21	1–9	0	
Mean Pack Years	28	20	41	0.3^1^
95% confidence interval	−6.2–62.2	−3.5–43.9	21.8–60.3	
Range	0−70	0–87.5	0–100	
Gender				1^2^
Male	5	6	8	
Female	0	3	2	
Stage				0.40^2^
IVA	4	7	9	
IVB	1	2	1	
Site				0.006^2^
Oropharynx	5	9	5	
Hypopharynx	0	0	4	
Larynx	0	0	1	

^1^ANOVA.

^2^Fisher's Exact Test.

**Table 2:** Tabulated scoring of the histologic analysis.

Parameter	Amifostine	Partial-amifostine	No-amifostine
Fatty replacement			
None	1	1	2
Trace	1	0	1
Mild	1	2	4
Moderate	2	6	2
Severe	0	0	1
Lobular architecture			
None	4	5	9
Mild	0	2	0
Moderate	1	2	1
Interstitial fibrosis			
None	0	1	3
Trace	0	0	0
Mild	4	6	6
Moderate	1	1	1
Severe	0	1	0
Ductal degeneration			
None	2	7	9
Mild	0	0	0
Moderate	3	2	0
Severe	0	0	1
Inflammatory component			
Diffuse	1	3	2
None	1	2	1
Trace	3	4	6
Mild	0	0	1
Moderate			
Focal			
None	2	6	6
Trace	0	1	1
Mild	2	2	2
Moderate	0	0	1
Acinar degeneration			
None	0	2	2
Trace	1	0	0
Mild	0	1	3
Moderate	3	5	4
Severe	1	1	1

**Table 3:** Histological variable analysis.

Parameter	*N*	*Q*1	Median	*Q*3	Cuzick's *P* value
Fatty replacement					0.82
Amifostine	5	1	2	3	
Partial-amifostine	9	2	3	3	
No-amifostine	10	1	2	3	
Lobular architecture					0.57
Amifostine	5	0	0	0	
Partial-amifostine	9	0	0	1	
No-amifostine	10	0	0	0	
Interstitial fibrosis					0.26
Amifostine	5	2	2	2	
Partial-amifostine	9	2	2	2	
No-amifostine	10	0	2	2	
Ductal degeneration					0.19
Amifostine	5	0	2	2	
Partial-amifostine	9	0	0	0	
No-amifostine	10	0	0	0	
Inflammatory component (diffuse)					0.55
Amifostine	5	1	2	2	
Partial-amifostine	9	0	1	2	
No-amifostine	10	1	2	2	
Inflammatory component (focal)					0.94
Amifostine	5	0	0	2	
Partial-amifostine	9	0	0	1	
No-amifostine	10	0	0	2	
Acinar degeneration					0.35
Amifostine	5	3	3	3	
Partial-amifostine	9	2	3	3	
No-amifostine	10	2	2.5	3	
